# Acoustic Actuators for the Manipulation of Micro/Nanorobots: State-of-the-Art and Future Outlooks

**DOI:** 10.3390/mi15020186

**Published:** 2024-01-26

**Authors:** Hiep Xuan Cao, Van Du Nguyen, Jong-Oh Park, Eunpyo Choi, Byungjeon Kang

**Affiliations:** 1 Robot Research Initiative, Chonnam National University, Gwangju 61186, Republic of Korea; cxhiep@jnu.ac.kr (H.X.C.); eunpyochoi@jnu.ac.kr (E.C.); 2 Korea Institute of Medical Microrobotics, Gwangju 61011, Republic of Korea; jop@kimiro.re.kr; 3 School of Mechanical Engineering, Chonnam National University, Gwangju 61186, Republic of Korea; 4 Graduate School of Data Science, Chonnam National University, Gwangju 61186, Republic of Korea; 5 College of AI Convergence, Chonnam National University, Gwangju 61186, Republic of Korea

**Keywords:** ultrasound, acoustic wave, acoustic actuator, microrobots, nanorobots, manipulation, biomedical applications

## Abstract

Compared to other actuating methods, acoustic actuators offer the distinctive capability of the contactless manipulation of small objects, such as microscale and nanoscale robots. Furthermore, they have the ability to penetrate the skin, allowing for the trapping and manipulation of micro/nanorobots that carry therapeutic agents in diverse media. In this review, we summarize the current progress in using acoustic actuators for the manipulation of micro/nanorobots used in various biomedical applications. First, we introduce the actuating method of using acoustic waves to manipulate objects, including the principle of operation and different types of acoustic actuators that are usually employed. Then, applications involving manipulating different types of devices are reviewed, including bubble-based microrobots, bubble-free robots, biohybrid microrobots, and nanorobots. Finally, we discuss the challenges and future perspectives for the development of the field.

## 1. Introduction

**Figure 1:**
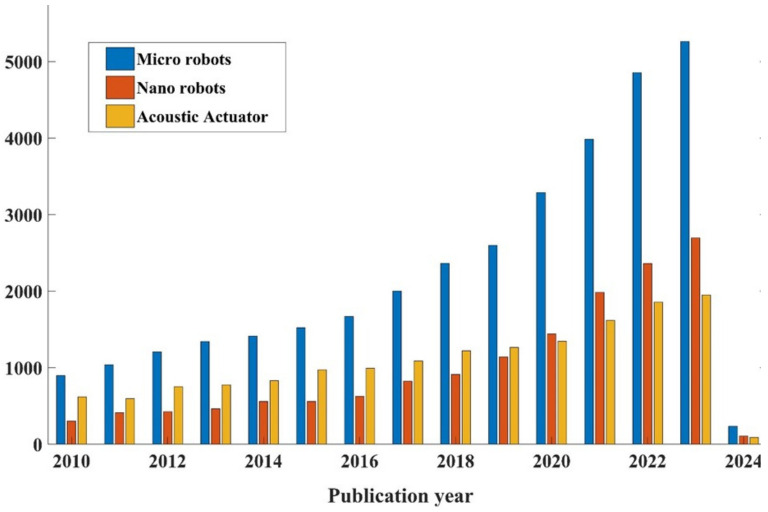
The number of scholarly articles featuring the keywords “micro robots”, “nano robots”, and “acoustic actuator” within the period from January 2010 to 15 November 2023, according to the Science Direct database.

The term “robot” originates from the Czech word “robota”, which means “forced labor”. It was initially introduced in 1921 in the play R.U.R. (translated as “Rossum’s Universal Robots”). Subsequently, Isaac Asimov (a Russian–American science fiction writer) portrayed robots as compassionate entities that fulfilled human needs and perceived them as an advanced and more refined species. Asimov proposed the “Three Laws of Robotics”, which governed his own robotic creations and influenced the depiction of robotic characters in various other science fiction narratives. Accordingly, the question of how to define a robot in real life is rather difficult to answer, although several definitions exist. In this review, we followed the definition proposed by the Robot Institute of America in 1979: a reprogrammable, multifunctional manipulator designed to move material, parts, tools, or specialized devices through various programmed motions for the performance of a variety of tasks. In the context of this review, the term micro/nanorobots (MNRs) refers to untethered robots ranging from nanometers to submillimeters. These robots are designed to autonomously perform specific tasks at the microscale or can be controlled externally. In contrast to conventional robots, MNRs can access very small areas that have never been observed previously, such as microchannels [1,2,3,4,5] and microvessels [6]. MNRs have demonstrated immense potential for utilization in a variety of biomedical applications, encompassing areas such as chemical detection [7,8], biosensing [9,10,11,12], and microsurgery. The rapid development of MNRs manufacturing technologies in recent decades has significantly propelled the evolution of autonomous robotic systems which have become valuable technologies for healthcare as well as in biotechnology, biomedical engineering, and life sciences. This advancement has opened up new possibilities for the application of these MNRs in various fields. Continuous research and development in this domain promise a future in which these MNRs can perform tasks that were once thought to be unachievable. One of the most promising applications is in the field of drug delivery [13,14,15,16,17]. In this context, MNRs serve as vehicles to transport therapeutic agents to the designated target sites, subsequently releasing the drugs. This targeted approach increases therapeutic efficacy and reduces the adverse side effects typically associated with systemic drug distribution. MNRs can self-propel through an internal or external energy source, such as chemical catalysis, light, electric/magnetic, and acoustic energy. Recent advancements in the fabrication of and measurement instruments used by MNRs have significantly broadened the application of these actuation techniques toward the self-propulsion of micro/nano-agents. In this review, we focused on controlling MNRs by using acoustic energy. According to the Science Direct database, the number of publications containing the keywords “micro robots”, “nano robots”, or “acoustic actuator”, shown in Figure 1, demonstrates an increasing interest in researching on MNRs and acoustic actuators from January 2010 to the date of this review (November 2023). Among the MNR power sources, acoustic energy has the unique characteristics of being contactless and biocompatible. Moreover, it is versatile and has a large range of working frequencies (from kHz to GHz). These characteristics have rendered acoustic energy a powerful platform for the wireless control of MNRs. In terms of biocompatibility, several studies have demonstrated that the operating parameters of acoustic sources can be optimized to avoid damage in cells and small-animal models. For example, red blood cells placed in an acoustic-tweezer device for the same duration do not exhibit developmental impairments or changes in mortality rates [18]. In terms of versatility, acoustic manipulation techniques can be employed to trap and maneuver MNRs as acoustic tweezers or enable fast fluid transport to pump and mix different liquids [19,20,21,22,23].

Many methods have been employed to actuate robots and move them to targeted areas, including biological, chemical, magnetic, and acoustic actuating. For biological actuating, the navigation of robots toward target sites relies on the inherent features of actuating biological cells. Biological actuating offers advantages such as biocompatible intrinsic power sources, the capacity to integrate sensing and targeting functions, and suitability for fluidic physical environments [24]. However, there are disadvantages, including a limited actuation force and relatively weak targeting ability. To improve the targeting effectiveness, it is recommended that additional targeting strategies are employed. Robots are chemically targeted through the induction of chemical reactions that produce bubbles to propel the robots. This propulsion mechanism relies on the catalytic decomposition of hydrogen peroxide, which is present in specific environments. This decomposition occurs through platinum nanoparticles coating the inner surfaces of the robots or their payloads, forming water and oxygen. This process enables the creation, formation, and release of oxygen bubbles from one end, instigating motion in the opposite direction [25]. While the chemical actuation method results in a higher induced propulsion velocity compared to other actuation methods, the locomotion is less directional and there are limitations in terms of targeting accuracy, quick action, and immediate feedback. Furthermore, the fuels which are essential for the robots are highly toxic, imposing limitations on the applicability of this targeting strategy. Magnetic targeting is highlighted as a potent and widely adopted technique for the precise control of robots within targeted tissues [26]. This method has various advantages, including untethered maneuverability, no fuel requirements, and compatibility with other systems. To respond to a magnetic field, robots must incorporate magnetic nanoparticles. Two common types of magnetic targeting employed for robot control are the electromagnet actuating (EMA) system and the permanent magnetic actuating (PMA) system. The EMA system comprises electromagnetic coils connected to power supplies that regulate the coil currents through computer software. This setup can produce a uniform magnetic field, magnetic gradient, and/or rotating magnetic fields to manipulate the robots. In comparison, the PMA system consists of a set of permanent magnets, and robot control is achieved by altering the distance and position of these magnets [27]. Each system possesses its own set of advantages and drawbacks. The EMA system enables the generation of a magnetic field in a three-dimensional space. Additionally, the magnetic current and frequency can be easily adjusted, providing flexible and precise control. The disadvantages of the EMA system include cost, space prerequisites, and heat generation. In contrast to the EMA system, the PMA system dispenses with the need for intricate coils, control interfaces, or power supplies, mitigating the overheating issue. Moreover, the induced magnetic flux density can be significantly higher. However, the magnetic field’s control flexibility is constrained, and halting the magnetic field during operation can prove challenging [28].

Targeting using acoustic actuators is emerging as a powerful technique for the manipulation of MNRs due to the generation of strong actuation forces, no physical contact with the robots, a biocompatible power source, and the possibility of combining them with ultrasound imaging to track the robots. Serval literature review articles have been published that provide a comprehensive understanding of microrobots [3,29,30,31,32,33], nanorobots [34,35,36], and acoustic actuators [36,37,38,39,40,41]. Each review provides researchers with a unique perspective and different approaches. In this work, we summarize the most current progress in using acoustic actuators for the manipulation of the MNRs for biomedical applications. The study commences by providing an overview of MNRs from fundamentals to applications. Additionally, the principles of acoustic control are described, which is the main field application of using acoustic control for MNRs in biomedical applications. Then, the applications involving manipulating different types of MNRs are reviewed, such as bubble-based microrobots, bubble-free microrobots, biohybrid microrobots, and nanorobots. Finally, the challenges and potential solutions for advancing MNR manipulation using acoustic signals are addressed.

## 2. Principle of Acoustic Manipulation

### 2.1. Acoustic Fundamentals

In the context of acoustic waves, a transducer is employed to initiate vibrations within a medium by converting electrical signals into mechanical oscillations, which are subsequently transmitted to the medium through a matching layer. An acoustic wave is a type of mechanical wave that travels through a medium, such as a solid, liquid, or gas. The properties of the medium through which the acoustic wave propagates play an important role in the regulation of the wave’s acoustic characteristics. For example, the speed of acoustic waves in solids is 6000 m/s. In liquid at 20 degrees Celsius, the speed of acoustic is 1481 m/s. The speed of acoustic in air is 343 m/s at 20 degrees Celsius, and at 0 degrees Celsius, it is 331 m/s.

There are four characteristics of acoustic waves: wavelength (m) is the distance between adjacent identical parts of an acoustic wave, frequency (Hz) is the number of wave that pass a fixed point in one second, velocity (m/s) is the speed at which a sound wave travels through a medium, and intensity (W/m^2^) is the ratio of the amount of acoustic energy carried by a wave per unit area in the direction perpendicular to that area [42,43]. The acoustic catalog can be classified based on the frequency range it covers, as follows:

- Infrasonic: These are acoustic waves with frequencies less than 20 Hz. They are typically used in applications such as seismic monitoring and for studying low-frequency sound phenomena.
- Sonic: This category includes acoustic waves with frequencies ranging from 20 Hz to 20 kHz, which is the range of human hearing. Sonic waves are widely used in various fields, including music, communication, and environmental noise analysis.
- Ultrasonic: Acoustic waves in the ultrasonic range have frequencies from 20 kHz up to 200 MHz. They find extensive use in medical applications, such as ultrasound imaging, non-destructive testing, and cleaning processes.
- Hypersonic: Acoustic waves with frequencies higher than 200 MHz fall into the hypersonic category. These waves are primarily used in acoustic microscopy, which enables high-resolution imaging of small-scale structures.

**Figure 2:**
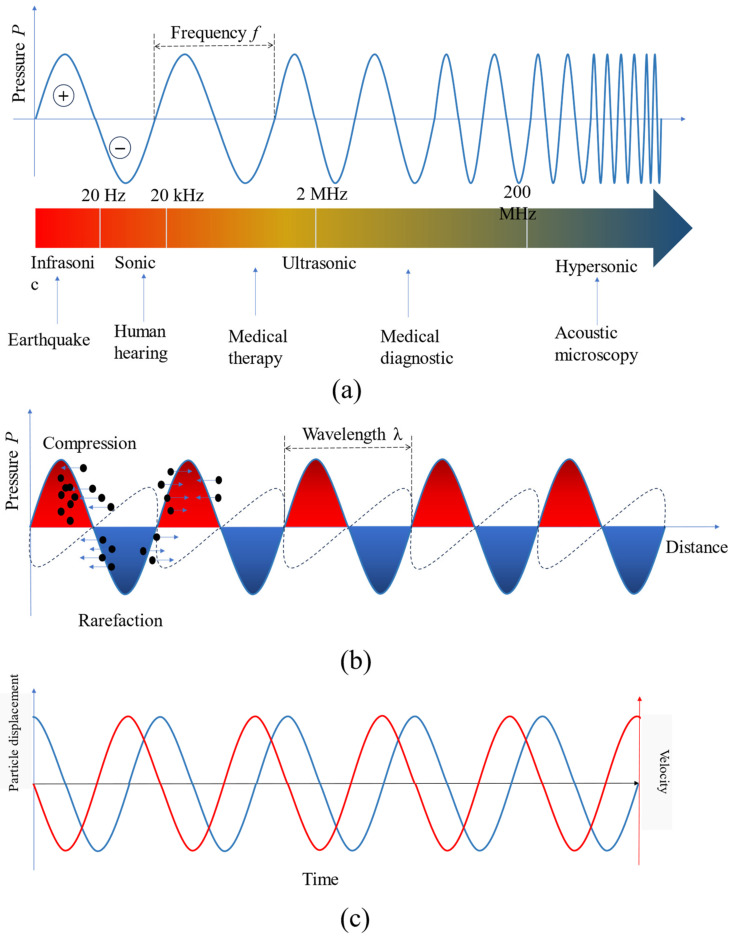
The fundamental characteristics of acoustic wave: (**a**) the acoustic catalog and its diverse applications; (**b**) the acoustic pressure field formed in the medium; (**c**) the particle displacement (blue color) and velocity (red color) in the time domain.

Figure 2a displays the acoustic catalog from infrasonic to hypersonic (less than 20 Hz to over 200 MHz) and its various applications.

Beyond the four fundamental parameters of acoustic waves, such as wave frequency, wave velocity, and wavelength, and acoustic intensity, a comprehensive characterization of an acoustic wave necessitates the consideration of several other crucial parameters. These include the displacement and velocity of particles, the absolute and oscillating acoustic pressures, fluctuations in density (which are a consequence of oscillating pressure), acoustic attenuation, and acoustic impedance. Acoustic pressure is a measure of the force exerted by an acoustic wave on a surface that is perpendicular to the direction of the acoustic source. It is measured in pascals (Pa). The oscillation of the particles in the medium which acoustic wave passes will generate a pressure field leading to periodic regions of compression (high pressure) and rarefaction (low pressure). This region is referred to as a pressure node. Objects submerged in the medium have a propensity to move towards these pressure nodes to achieve stability. Figure 2b shows the compression area and rarefaction area referring to the acoustic wavelength. This fundamental property of acoustic waves can be utilized to manipulate the target within an acoustic pressure field. Figure 2c shows the dependence of particle displacement on velocity. The phase difference between the acoustic pressure and velocity is 0 degree or 180 degrees, the phase difference between velocity and displacement is 90 degree or −90 degrees depending upon the coordinates reference and direction of wave propagation. One more acoustic parameter we should consider is acoustic impedance, which measures a material’s resistance to the propagation of an acoustic wave; it is determined by the product of the speed of sound and the density of the medium.

### 2.2. Acoustic Radiation Force

Sound waves traveling through a gas or liquid create an acoustic field that generates varying acoustic pressures. These pressure differences in the acoustic field can exert forces on objects that are either immersed or suspended, which are commonly known as acoustic radiation forces. Additionally, sound waves induce motion in the surrounding medium by means of a phenomenon known as acoustic streaming.

**Figure 3:**
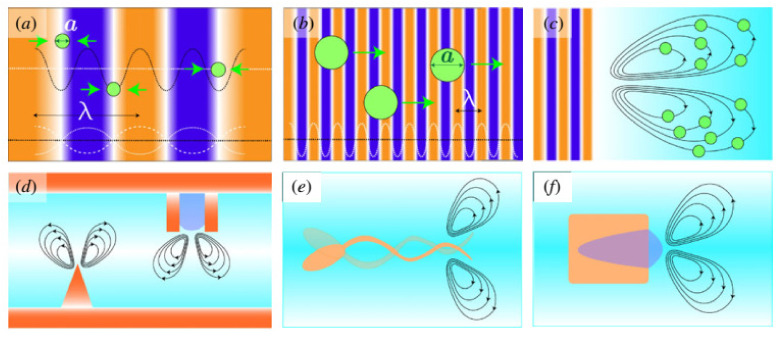
Acoustic radiation forces acting on target object with dimension *a* and acoustic source of wavelength *λ*, where *k* = 2*π*/*λ* is defined as the ratio between 2*π* and wavelength. Scenario (**a**): dominant force acting on target object is gradient force that results in potential acoustic field U; scenario (**b**): scattered forces overcome the gradient force, which is most dominant force acting on target object under traveling wave propagation; scenario (**c**): drag force is most dominance force acting on target object cause by streaming; scenario (**d**): streaming patterns formed by vibration; scenario (**e**): asymmetrically designed micro swimmer; and scenario (**f**): bubble self-propulsion. Reprinted from ref. [36]. Copyright 2020 the Author(s).

In the acoustic field, the immersed or suspended object will be affected by four types of force: *F_g_* is the gravitational force acting on the object, *F_rad_* is the acoustic radiation force, *F_scatter_* is the acoustic scatter forces, and the *F_stream_* is the force on an object caused by the stream. Figure 3 displays the acoustic radiation forces acting on a target object and its dimensions. In summary, the movement of an object in an acoustic field is described by the following equation:

(1) $$m\ddot{x}=F_{g}+F_{rad}+F_{scatter}+F_{stream}$$

Scenario ka≪1 refers to King’s study on “Acoustic radiation pressure on a compressible sphere”, [44] the result of which was subsequently confirmed by L. P. Gor’kov in 1962 [45]. In summary, the resulting radiation force acting on small particles in the scenario ka≪1 can be calculated as the gradient of acoustic potential U following Bruus. H “Acoustofluidics 7” [46]. Thus, the acoustic radiation force (*F_rad_*) can be written as

(2) $$F_{rad}=-\nabla U,$$

where potential field *U* is

(3) $$\nabla^{2}U=\frac{\partial^{2}U}{\partial^{2}x}+\frac{\partial^{2}U}{\partial^{2}y}+\frac{\partial^{2}U}{\partial^{2}z},$$

(4) $$U=2K_{1}\left({\left|p\right|}^{2}\right)-2K_{2}\left({\left|p_{x}\right|}^{2}+{\left|p_{y}\right|}^{2}+{\left|p_{z}\right|}^{2}\right).$$

The scattering coefficients *K*_1_ and *K*_2_ are calculated as follows:

(5) $$K_{1}=\frac{1}{4}V\left(\frac{1}{c_{0}^{2}\rho_{0}}-\frac{1}{c_{1}^{2}\rho_{1}}\right),$$

(6) $$K_{2}=\frac{3}{4}V\left(\frac{\rho_{0}-\rho_{1}}{\omega^{2}\rho_{0}\left(\rho_{0}+2\rho_{1}\right)}\right),$$

where *V* is the volume of the particle, *ω* is the angular frequency of the emitted acoustic wave, *ρ* is the density of the target object, and *c* is the speed of sound in the medium (with subscripts 0 and 1 referring to the host medium and the particle material, respectively). Term |p| is the mean value of the complex pressure and its derivative in Cartesian x-, y-, and z- coordinates as $\left|p_{x}\right|,\;\left|p_{y}\right|,\;\left|p_{z}\right|$*,* respectively.

In the scenario *ka* < 1, the most dominant force acting on the target agent is the streaming-induced force. The total force is obtained by linearization using the perturbation approximation to estimate the Stoke drag force. The drag force is written as follows [47]:

(7) $$F=6\pi \eta r(\left\langle v_{2}\right\rangle -v_{1})$$

where η is the viscosity of medium, and the target object radius *r* moving with velocity *v*_1_ in the medium has the streaming velocity $\langle v_{2}\rangle$.

In the scenario *ka* ≥ 1, the scattered forces are the most dominant force. The acoustic radiation force on the larger spherical target agent is calculated based on the second-order acoustic pressures around a boundary that encloses the object [48]:

(8) $$\vec{F}=-\langle \int_{s}p_{2}\vec{n}dS\rangle -\langle \int_{s}\rho_{0}(\vec{n}.{\vec{v}}_{1}){\vec{v}}_{1}dS\rangle$$

where *S* defines the boundary that encloses the object and $\vec{n}$ is the normal of the defined surface *S*. Some researchers have developed self-propelled MNRs by vibrating the air bubbles that drive MNRs in the acoustic region [49,50,51,52]. In these studies, the bubble vibrated at its resonant frequency (*f*_0_), which depended on the geometry of their air-filled parts to drive the target agent. The resonant frequency of the bubble can be expressed as follows [53]:

(9) $$f_{0}=\frac{1}{2\pi }(\sqrt{\frac{\kappa P_{0}}{\rho L_{0}L_{B}}})$$

where κ represents the frequency-dependent parameter, *P*_0_ is the ambient pressure in the acoustic medium, *L_B_* is the length of the bubble, and *L*_0_ is the total length of the liquid column between the surface of the bubble and the exit of the cylindrical cavity.

### 2.3. Acoustically Actuated Micro/Nanorobot Strategy

In this study, we investigated various strategies for acoustic manipulation in relation to the previously mentioned theory of radiation and streaming-induced forces. Acoustically actuated MNRs can be classified into two primary areas by examining the working principle of the actuation: acoustic tweezer actuation and autonomous propelling acoustic actuation. Table 1 lists the main characteristics of each acoustic actuation strategy, including the propelling mechanism, the acoustic control type, and possible applications.

#### 2.3.1. Acoustic Tweezer Propulsion

The working principle of acoustic tweezer propulsion is to generate a geometric acoustic field within a defined workspace. Within this geometry, the acoustic gradient forces act on target objects and can be controlled to trap them in the desired location. By repositioning or adjusting the acoustic tweezer, the motion of the target objects can be controlled and manipulated to follow the movement of the acoustic tweezer. The two primary types of acoustic tweezer propulsion based on the characteristics of the acoustic propulsion mechanism are standing- and traveling-wave tweezers.

- Standing-wave tweezers

To generate tweezers using a standing acoustic wave, the acoustic waves are generated from a transducer. Different types of transducers have varying operating principles. Standing acoustic waves can be further categorized into two subtypes: surface acoustic waves (SAWs) and bulk acoustic waves (BAWs). SAWs are generated on the surface of a piezoelectric film and are created by interdigital transducers (IDTs) fabricated on the surface of a transducer. The characteristics of SAWs, such as resonance frequency, and amplitude, are identified by the characteristic designs of the IDTs, such as the electrode dimensions, material properties, and applied electrical signal [56,74]. In the fabrication process, IDTs are generally made from microscopic devices incorporating both electronic and moving parts. The components can be a very small size of up to 1 micrometer. Thus, SAW-based tweezers can be easily integrated with microfluidic systems, making them a versatile tool for lab-on-a-chip applications.

BAWs are produced by applying an alternating (AC) electrical signal across both sides of a piezoelectric material. This causes an acoustic wave to propagate throughout the entire thickness of the material, resulting in the formation of a stationary wave at specific frequencies [75]. When acoustic waves are reflected from the reflection layer, they create standing waves that establish a pressure distribution in the fluid. By adjusting the frequency of the waves in relation to the dimensions of the channel’s geometry, it is possible to customize the number of pressure nodes and antinodes formed within the channel.

Owing to the high-precision acoustic patterns, standing-wave tweezers are primarily utilized in applications for the separation and arrangement of various particles and cells. BAW-based standing-wave tweezers offer the advantage of handling larger fluid volumes within a shorter duration, making them desirable for applications such as blood processing in transfusion scenarios [76,77,78]. In contrast, SAW-based tweezers exhibit higher precision due to their utilization of higher frequencies, making them more suitable for nanoparticle manipulation and tissue-engineering applications [54,57,79,80,81,82,83].

- Traveling-wave tweezers

Traveling-wave acoustic tweezers generate acoustic pressure nodes through the deliberate design of a singular beam structure, instead of relying on beam interference. This is typically realized through the meticulous calculation of phase patterns across the radiation aperture [61,84,85,86,87,88,89,90,91,92]. The initiation of an acoustic wave can emanate from either a solitary element or an array comprised of multiple elements, forming traveling-wave tweezers. In the case of a single element, an acoustic lens is positioned on the element’s surface. Manipulation of the acoustic focusing field is facilitated through the geometric design of the acoustic lens. This approach enables the generation of a singular traveling-wave acoustic tweezer conforming to pre-established parameters. The targeted object will be trapped in a stable tweezer. To make the targeted object move, the whole structure (including the transducer and lens) is moved. This characteristic represents a constraint which is inherent in traveling-wave tweezers derived from a singular acoustic element. To overcome this limitation, an acoustic wave can be generated through an array composed of multiple elements. Employing a phase modulation control algorithm in this approach enables the calculation of distinct delay times for the electric signal directed to each element. Through the determination of appropriate delay times, many types of tweezer configurations can be established. The three most popular types of traveling-wave tweezers resulting from this method are the twin tweezer [17,62,93], bottle tweezer [51], and vortex tweezer [61,63,94]. Compared to standing-wave tweezers, traveling-wave tweezers are capable of greater real-time modulation and are proven to be more suitable for in vivo applications.

#### 2.3.2. Streaming-Driven Acoustic

An acoustic stream is a steady fluid flow generated by the nonlinear interaction of acoustic waves with a fluid medium, usually involving vibrating micro air bubbles or a vibrating solid boundary. These streams are intended to control both the surrounding medium and any MNRs that are immersed in the medium. Based on the source of stream generation, it can be classified into two types: (1) streams based on bubble oscillations and (2) streams based on the vibration of the solid boundaries applied in geometric designs. In streams based on bubble oscillations, the bubbles oscillate at a natural frequency to create surrounding fluid vortices. Hence, they can rotate MNRs in a fixed position and enable fluidic actuation by enhancing mass transport across the laminar flows in confined microchannels [95,96,97]. In streams based on the vibration of a solid boundary, the acoustic wave is reflected on a solid boundary to create fluid flows along the boundary. The geometry of the device is designed with either sharp protrusions or by incorporating symmetric structures. The solid boundary can be a rigid wall, a flexible membrane, or a microstructure. Although stream-driven acoustics require a simple acoustic actuator that is user-friendly, they are restricted to use in a liquid medium and have a lower degree of spatial resolution. Therefore, this technique is primarily intended for employment in microfluidic channel technology, fluid management, or the rotational manipulation of biological samples [21,66,97].

## 3. Acoustic Manipulation of Micro/Nanorobots

Acoustics can function within a frequency range of kilohertz to gigahertz, facilitating the capture and manipulation of target objects spanning a size range from nanometers to centimeters [6,20,98,99,100,101,102,103,104,105,106,107,108,109,110,111,112,113]. Over the past decade, global researchers have made substantial advancements in the acoustic manipulation of micro/nano robots (MNRs). These developments are on the cusp of transitioning into clinical applications. Huang’s group developed an acoustics platform implemented with microfluidics which can isolate 110 nm particles from a complex culture media including micro and nano sized different particles with a yield greater than 99% [114]. The SAWs tweezer was used to separate and analyze circulating tumor cells (CTCs) from whole blood cells (WBCs) with a recovery rate better than 83% [115]. Cao et al. introduced AcoMan, an acoustic manipulation device designed for the manipulation of nanoclusters in water with five degrees of freedom (5-DoF) [17,62,93]. The transducer array is composed of 30 ultrasonic elements operating at 1 MHz, with an operating voltage of 60 Vpp. AcoMan exhibits the capability to manipulate nanoclusters within a working space of 5 × 5 × 4 m, achieving a position error of less than 200 μm. Furthermore, it facilitates the rotational movement of clusters without vertical restrictions and within a horizontal range spanning from −30.31° to +29.93°. Ghanem et al. developed a steerable, vortex-based acoustic trapping beam which can lift and move a 3 mm glass sphere inside a pig’s bladder [94]. The system can perform 3D movement with less than a 10% position error compared with the intended trajectory. No injuries were detected on the bladder wall or the intervening tissue. 

In this section, the acoustic manipulation of different types of robots is discussed, including bubble-based microrobots, bubble-free microrobots, biohybrid microrobots, and nanoparticle-based robots (nanorobots).

### 3.1. Bubble-Based Microrobots

**Figure 4:**
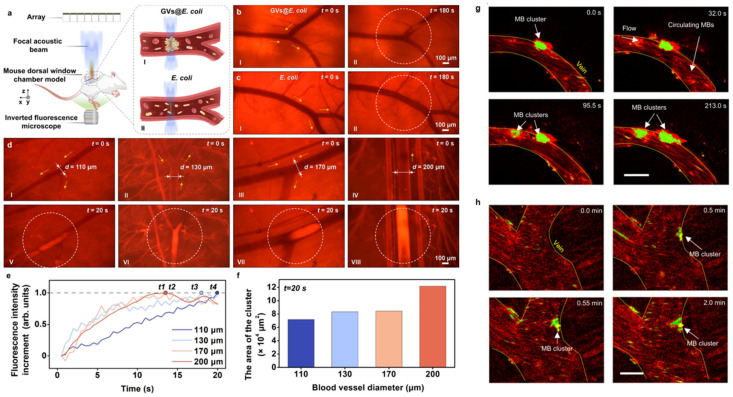
(**a**) Schematic diagram of in vivo experiment setup. I and II are schematic diagrams of the acoustic trapping processes of the GVs@*E. coli* and control *E. coli*, respectively. Comparison of acoustic trapping of (**b**) GVs@*E. coli* and (**c**) control *E. coli* in superficial blood vessels on the backs of mice. I in (**b**,**c**) indicates the microscopic images of blood vessels in the absence of ultrasound after injection of GVs@*E. coli* and control *E. coli*, respectively. II in (**b**,**c**) shows the microscopic images of blood vessels after exposure to ultrasound for 180 s based on the situation I in (**b**,**c**), respectively. Only GVs@*E. coli* can be trapped at the focal beam center and form clusters in the vessels. (**d**) Acoustic trapping of GVs@*E. coli* in blood vessels of different diameters. I, II, III, and IV are microscopic images of 110, 130, 170, and 200 μm diameter vessels injected with GVs@*E. coli* before the ultrasound is on, respectively. V–VIII are microscopic images of trapped GVs@*E. coli* clusters in the corresponding blood vessels in cases I–IV after the ultrasound is turned on for 20 s. The yellow arrows, white dotted circles, symbol (**d**), and t in (**b**–**d**) indicate the blood flow direction, focal zones, vessel diameter, and time, respectively. (**e**) The curves of normalized fluorescence intensity increment over time within the focal zones under vessels of different diameters in (**d**). t1 to t4 represent the moment when the maximum increment of fluorescence intensity is reached in 200, 170, 130, and 110 μm diameter vessels, respectively. (**f**) The area of the GVs@*E. coli* cluster in vessels of different diameters (after the ultrasound is turned on for 20 s). Reprinted from ref. [116] under the terms of the Creative Commons Attribution 4.0 International License. Copyright 2023 the Author(s). (**g**,**h**) Microbubbles (MB) aggregate over time and adhere to the walls of venules under an acoustic signal of 490 kHz and 35 V_PP_. This phenomenon was reproduced in at least ten independent acoustic activation experiments. After image processing, the microswarms were colored in bright green and marked with an arrow while non-responding single microbubbles that flowed downstream were colored in red. Scale bars are 50 µm. Reprinted from [117] under the terms of the Creative Commons Attribution 4.0 International License. Copyright 2023 the Author(s).

Normal living cells are difficult to manipulate with an acoustic actuator due to the cells having a similar acoustic impedance to the medium. To solve this problem, Yang et al. used genetic engineering on bacteria to ensure that many gas bubbles of sub-micron size could be induced in the bacterial cytoplasm, enabling the engineered bacteria (GVs@*E. coli*) to be sensitive to and manipulated by acoustic waves [116]. Using a 64-element acoustic tweezer operating at 3 MHz, the authors steered the GVs@*E. coli* under a counter flow or on-demand flow in the vasculatures of live animals. In addition, the tumor accumulation of the bacteria was improved after acoustic guidance (Figure 4a–f). Incorporating microbubbles into the microrobots enabled them to be manipulated by an acoustic actuator due to the interaction of the microbubbles with the acoustic wave. In a recent study, Fonseca et al. prepared microrobots consisting of lipid-shelled microbubbles that had the ability to autonomously aggregate and be propelled when exposed to ultrasound irradiation [117]. Using piezo transducers of 3 × 3 mm at a resonance frequency of 490 kHz, the authors could control and track the microrobots in vitro using artificial vasculatures and in vivo within the brain vessels of a live animal with a moving velocity of up to 1.5 μm/s and against a blood flow of approximately 10 mm/s (Figure 4g,h).

In addition, microbubbles can be incorporated into 3D-printed microstructures to allow effective manipulation using acoustic actuators [118,119,120,121,122]. This is possible because the confinement of microbubbles within the microstructure’s cavities results in significant scattering, resulting in robust propulsive forces when activated by acoustic waves [123]. 3D printing using two-photon lithography (TPP) is an emerging technology widely adapted to fabricated micro/nanoscale robots. TPP allows facile and precise fabrication of robots down to the nanoscale. In addition, the shape and size of the robot can be customized using commercial design software.

### 3.2. Bubble-Free Microrobots

**Figure 5:**
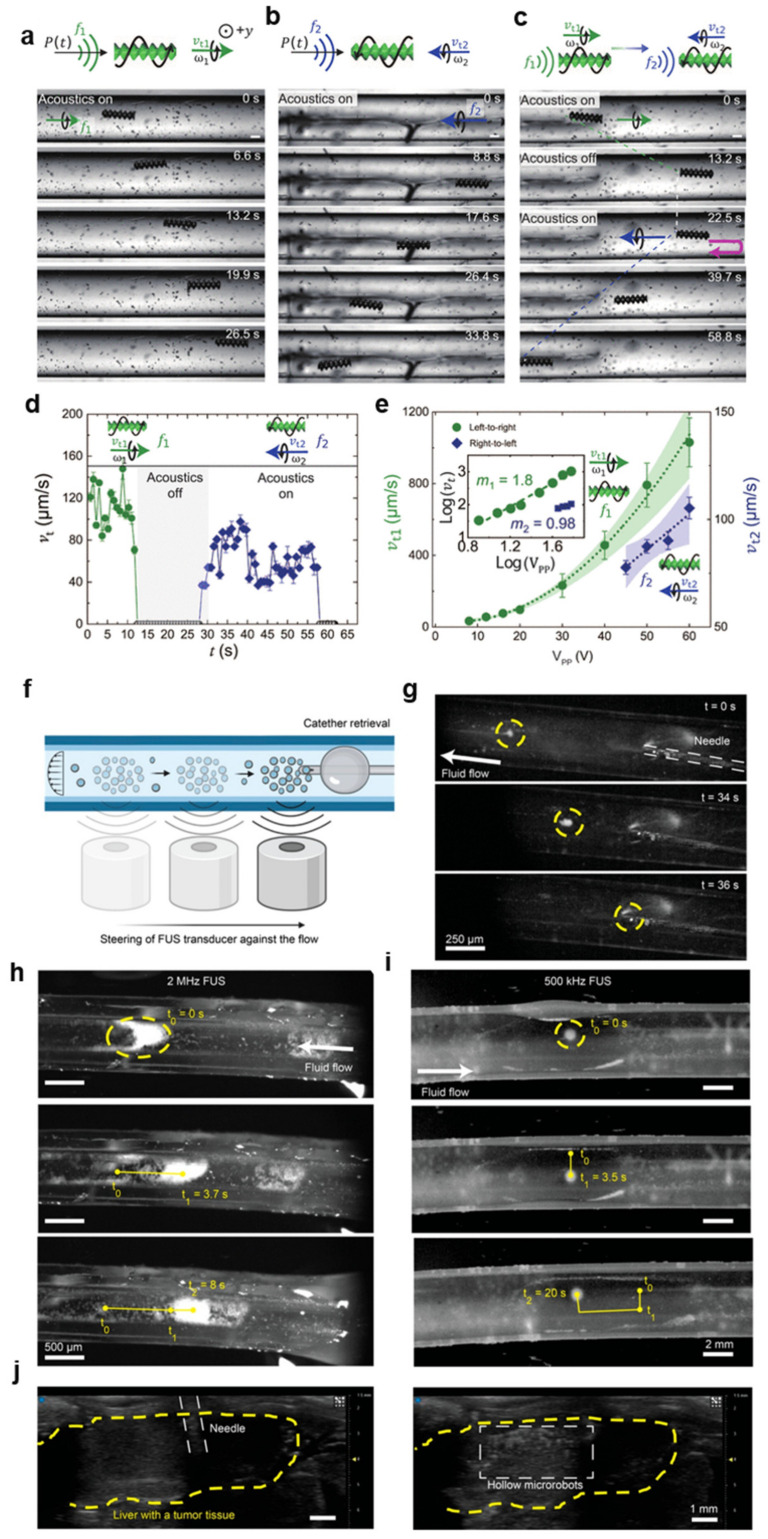
(**a**–**e**) Translational motion of the microrobot in a circular microchannel. (**a**) The microrobot exhibits left-to-right locomotion in response to an external acoustic wave at f1 = 13.5 kHz and 20 V_PP_. The symbol P(t) in the top schematic represents the pressure wave, while the black arrow indicates the direction of the acoustic wave field. (**b**) The microrobot exhibits right-to-left locomotion for f2 = 18.6 kHz and 60 V_PP_. (**c**) A microrobot illustrates its bidirectionality when switching from f1 = 13.5 kHz and 20 VPP to f2 = 18.6 kHz and 60 V_PP_. (**d**) The plot illustrates the speed profile of the microrobot during its bidirectional trajectory. (**e**) The microrobot’s left-to-right velocity vt1 and right-to-left velocity vt2 versus the acoustic driving voltage V_PP_ indicate that the microrobot propels at a speed nearly proportional to V^2^_PP_. The inset displays the corresponding log-log plot. Note that the scaling of vt2 was difficult to predict because of a lack of data points at higher voltages, as the maximum voltage was restricted by our amplifier to 60 V_PP_. Scale bars, 100 μm. The direction of gravity is antiparallel to the y direction, i.e., perpendicular to the figure plane. Each data point represents the average velocity of at least three microrobots. The error bar represents the SD. Reprinted from [123] under the terms of the CC BY-NC license. Copyright 2023 the Authors. (**f**,**j**) Control and retrieval of the hollow particles against the fluid flow. (**f**) Schematic representation of hollow particles retrieved using acoustic manipulation enabled by a FUS transducer against the fluid flow. (**g**) Time-lapse images showing the retrieval of the hollow microparticle swarm immersed into a 4.77 mm-diameter Tygon tubing against a flow speed of 0.93 mm/s. The particles are trapped by using a 500 kHz focused ultrasound transducer. The particles are moved back to the injection side by using a relative movement between the tubing and the transducers. There they are retrieved using the same 28 G needle used for injection. (**h**) Movement of acoustically trapped hollow microparticles inside a 500 μm diameter tubing. Here, 100 μL of particles with a concentration of 5 mg/mL are injected into a tubing using a 28 G needle. After injection, the particles are trapped using a 2 MHz focused ultrasound transducer and moved inside the tubing. The particles are imaged using a high-speed camera mounted to a light microscope. The applied flow speed is 25.46 mm/s. (**i**) The particles are trapped using a focused 500 kHz transducer. Using a manual x-y-z stage the particles were moved inside the tubing in 2D. The applied flow speed was 4.67 mm/s. (**j**) Ultrasonographic images of needle positioning (**left**) and subsequent particle injection (**right**) into the portal vein of the liver of an ex vivo mouse with multiple liver metastasis. The liver area is marked in yellow. Ultrasound imaging is performed in the B-mode with a frequency of 40 MHz. Reprinted from Ref. [125] under the terms of the CC-BY 4.0 license. Copyright 2023 the Authors.

Using microbubbles encapsulated in the microstructures provides an efficient approach for the manipulation of microrobots using an acoustic transducer. However, the performance of the microrobots is not stable and is only reliable for a couple of hours due to the short lifetime of the microbubbles being carried, which initiate a gradual switch in the operating resonance frequency [124]. In addition, to retain the microbubbles inside the microstructures, advanced hydrophobic treatment is needed. These problems limit the use of bubble-based robots for in vivo applications. Therefore, bubble-free robots with sharp-edged structures that allow steady streaming in liquids when excited by acoustic waves are currently gaining attention from researchers to address the limitations of bubble-based robots. For instance, in their recent study, Deng et al. used TPP to print a helical microrobot with dimensions of 350 μm in length and 100 μm in diameter and demonstrated that the microrobot was capable of locomotion through a fin-like double-helix microstructure [123]. Specifically, the microrobot exhibits responsive movements to sound stimuli within the frequency range of approximately 13.5 to 18.6 kHz, mimicking the spiral motion observed in natural biological bacteria. The asymmetric helical structure interacts with the incident acoustic field, generating a propulsion torque that produces rotation around the long axis of the microrobot. Interestingly, the microrobot has the unique ability to switch directionality simply by tuning the frequency of the acoustic field. Using a single acoustic piezoelectric transducer connected with a function generator and an amplifier, the authors demonstrated locomotion in both 2D and 3D artificial vasculatures (Figure 5a–e). Most recently, Wrede et al. proposed the utilization of hollow borosilicate microparticles characterized by a rigid thin shell, which can be effectively trapped and manipulated using a single-lens focused ultrasound transducer (frequency of 2 MHz or 500 kHz) under physiologically relevant flow conditions [125]. These hollow microparticles provide stability and advantageous acoustic properties. The authors demonstrated the successful trapping dynamics of the transducer within circular tubing of varying diameters, confirming the effectiveness under realistic flow rates and ultrasound amplitudes. Additionally, they illustrated the capability to displace hollow microparticles by steering the transducer against the flow. Moreover, potential biomedical applications were also presented, including active cell tagging and navigation in bifurcated channels, as well as ultrasound imaging in mouse cadaver liver tissue (Figure 5f–j).

### 3.3. Biohybrid Microrobots

**Figure 6:**
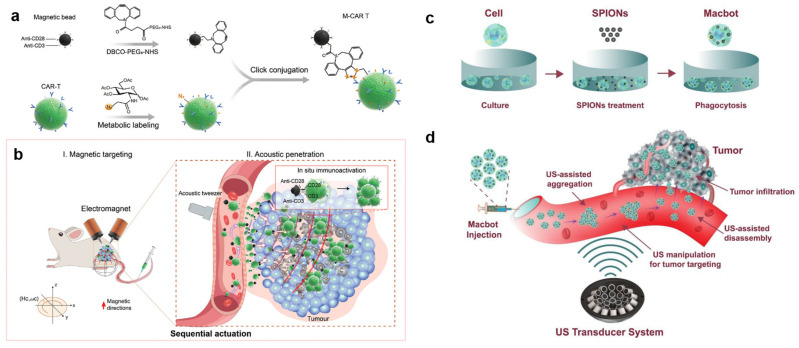
Schematic illustration of magnetic–acoustic sequentially actuated M-CAR Ts for programmable solid tumor targeting and enhanced immunotherapy. (**a**) Immunomagnetic beads were modified with dibenzocyclooctyne (DBCO) motifs using DBCO-PEG5-NHS conjugation, and azide-labeled CAR T cells (N3-CAR T) were obtained by simultaneous cell glycometabolic labeling with azido sugar Ac4GalNAz. Next, N3-CAR T cells were effectively conjugated with DBCO-modified beads to manufacture functionalized M-CAR Ts by click chemistry between DBCO and azide groups. (**b**) Magnetic–acoustic sequential actuation successfully achieved precision targeting and in situ immunotherapeutic activity of M-CAR Ts in vivo. Step I (**left**): intravenously injected M-CAR Ts precisely targeted and accumulated at peritumoural tumor margins driven by an external gradient magnetic field. The red arrows indicate the magnetic direction. Step II (**right**): peritumoural-accumulated M-CAR Ts were propelled by acoustic force from tumor-fixed acoustic tweezers to migrate deeply into the deep tumor. Finally, immunomagnetic beads modified with anti-CD3/CD28 in situ stimulated the proliferation and activation of CAR T cells, thus achieving a robust antitumor effect. Reprinted with permission from Ref. [127]. Copyright 2023 Wiley-VCH GmbH. Schematic diagram showing the concept of using acoustically driven microrobots for targeted tumor therapy: (**c**) fabrication process of the microrobots; (**d**) working principle of the microrobots using ultrasonic actuator system. Reprinted from ref. [134] under the terms of the CC-BY license. Copyright 2022 the Authors.

Biohybrid microrobots are prepared by hybridizing living cells with synthetic materials. The cells can be either immune cells (such as monocytes/macrophages [126], T cells [127], neutrophils [128]), red blood cells [129], or bacteria [130]. The synthetic materials are usually encapsulated with drugs or therapeutic agents as accompanying payloads for the robots. Combining the cells and payloads is performed by either internalization (endocytosis) [131] or surface attachment via chemical binding [132]. Wu et al. designed a hybrid microrobot using the functionalization of magnetic nanoparticles (MNPs) into red blood cells (RBC). The asymmetric distribution of the MNPs in the RBC induced a net magnetization, allowing the microrobots to be manipulated by both a magnetic field and acoustic propulsion [133]. Using a piezoelectric transducer mounted to a glass slide with a continuous sine wave operating at 2.93 MHz and a voltage amplitude tuned in the range of 0 and 10.0 V, the microrobots could reach a velocity of over 30 μm/s in PBS. Tang et al. [127] prepared a chimeric antigen receptor T cell robot (M-CAR T) by conjugating magnetic beads with CAR T cells (Figure 6a,b).

The M-CAR T was controlled sequentially by an electromagnetic field and an acoustic tweezer comprising a 3 MHz, 64-element (8 × 8) array, which was able to generate a focusing vortex acoustic field using a maximum peak-to-peak acoustic pressure of the vortex of 1.58 MPa at an excitation voltage of 30 V [127]. Due to the dual actuation in vivo, the authors verified that the accumulated CD8+ CAR T cells increased 6.6-fold compared to the number of cells without the actuation. As a result, the in vivo antitumor efficiency was enhanced in a CD19-SPCA1 tumor-bearing mouse model. Recently, our group used a newly designed acoustic actuator to manipulate macrophage-based biohybrid microrobots to propose a feasible approach to actively target tumors for antitumor therapy [134]. The acoustic actuator comprised 30 ultrasonic transducers operating at a frequency of 1 MHz, generating a twin-trap configuration at a trapping point. These microrobots were prepared by hybridizing macrophage cells with magnetic nanoparticles that functioned as therapeutic payloads of the microrobots. When exposed to an acoustic field, a group of microrobots could be precisely guided along a predetermined path, enabling them to approach the target from various perspectives.

Consequently, we successfully controlled the microrobot cluster to reach any point within a 4 × 4 × 4 mm region of interest, with an impressive position error of under 300 µm. Moreover, the cluster demonstrated the ability to rotate within the O-XY plane at 45° intervals with no constraints on the overall range of angles (Figure 6c,d).

### 3.4. Nanorobots

**Figure 7:**
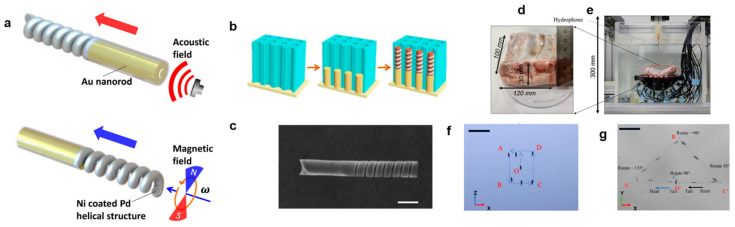
(**a**) Scheme of the design of the magneto–acoustic hybrid nanomotor and its dual propulsion modes under acoustic and magnetic fields. (**b**) Schematic illustration of the template-assisted fabrication of the bisegment magneto-acoustic hybrid nanomotors. (**c**) SEM image of a magneto–acoustic hybrid nanomotor. Scale bar: 500 nm. Reprinted with permission from Ref. [135]. Copyright 2015 American Chemical Society. (**d**–**g**) Ex vivo experiments for testing the manipulation and rotation of nanocarrier cluster: (**d**) porcine rib dimension; (**e**) Experimental setup; (**f**) time-lapse images of the manipulation in the XOZ planes; (**g**) time-lapse images of the manipulation and rotation in the XOY plane (scale bar: 2 mm). Reprinted from ref. [17] under the terms of the CC-BY license. Copyright 2022 the Authors.

Although not widely investigated in the literature, the feasibility of using an acoustic actuator to manipulate nanorobots has been demonstrated. In a pioneering study, Li et al. prepared a hybrid nanorobot (nanomotor) comprising a concave gold (Au) nanorod at one end and a nickel (Ni)-coated palladium (Pd) helical nanospring at the other end (Figure 7a–c). The concave Au nanorod structure was used for efficient acoustic manipulation, whereas the helical Ni-coated nanospring enabled the nanorobot to be controlled within a magnetic field [135]. Thus, the nanorobots could switch their operation modes by switching the acoustic and magnetic signals. Under acoustic manipulation, the nanorobot could reach a speed of 22.3 μm/s at an applied voltage of 6 V. In a recent study, we used an acoustic transducer system with 30 ultrasound transducers with a 1 MHz operating resonance frequency and controllable peak-to-peak voltage of 10 to 200 V, which were arranged on a single side to generate active traveling waves [17]. These waves were utilized to manipulate the position and orientation of untethered nanorobots within a spherical workspace in water, allowing for three degrees of freedom translation and two–three degrees of freedom rotation. A phase modulation algorithm was applied to independently control the phase signal for each of the transducers, enabling the precise manipulation of the positions of the nanorobots. The system utilized phase modulation and a switching power supply for each transducer to achieve rotation of the nanorobots in the horizontal plane. Additionally, the amplitude of the power supply to each transducer was controlled to facilitate rotating the nanorobots in the vertical plane. The viability of this method was demonstrated through in vitro and ex vivo experiments involving porcine ribs (Figure 7d–g) and Table 2 shows the different types of acoustic actuators for the manipulation of MNRs and applications

## 4. Challenges and Future Outlooks

As a promising method for manipulating MNRs, acoustic manipulation remains at an immature stage of development, although it has been applied to many fields of research in biomedical applications. However, many challenges remain within this technology in biomedical applications, the two main categories of which can be highlighted based on the acoustic propulsion source and the following MNR aspects: (1) Micro/nanorobotics is an emerging interdisciplinary field that encompasses various scientific domains including mechanical, electronic, material, biology, and other fields. Accordingly, realizing the potential applications of MNRs in the biomedical sector necessitates the collaborative efforts of researchers from these diverse disciplines to surmount the inherent challenges. (2) In terms of the current technology for fabricating MNRs, there are still very limited material choices available. Consequently, potential biomedical applications are limited by the MNRs’ materials. (3) The biodegradability and biological circulation of these materials remain challenging in practical biomedical applications.

The problems with the aspect of acoustic source propulsion are as follows: (1) The key challenge in actuating MNRs using acoustics is using them with complex media, such as the human body. The difference in acoustic impedance between various materials that the acoustic energy needs to pass through before reaching the target can dramatically reduce its energy and even change its properties. This issue is a significant obstacle that needs to be addressed to make the acoustic actuation of MNRs more effective. (2) Currently, the second main challenge in using acoustic actuation is the limitation of acoustic actuators’ spatial and temporal resolution. By using higher frequencies, although the target control is smaller, the acoustic penetration is reduced. Thus, many researchers are studying metamaterials and phononic crystals to improve the precision of the acoustic actuator. (3) In biomedical applications, safety and causing no harm to the target are most important. However, acoustic energy can harm the target through the generated heat and acoustic cavitation. To avoid these problems, the standardized methods to characterize the influence of acoustic waves on specific organs and frequency need to be researched and open accessed. From this, researchers could guarantee that the technology is compatible with their research goals.

In addition, most extant research has employed single or arrays of a small number of commercial piezoelectric actuators that induce a small actuating force and could limit the flexibility and working space of the actuating system. This reduces the applicability of acoustic actuating systems in comparison to other types of systems. Therefore, future research should focus on developing an acoustic actuating system with a stronger actuating force, larger working space, and should include an imaging function. In terms of manipulating function, although the feasibility of manipulating MNRs has been well proven in the reviewed literature, directional controllability remains challenging. Recently, Deng et al. proved the possibility of directionally controlling helical microrobots in simple 3D channels with different angles of inclination [123]. Thus, for efficient directional manipulation of the microrobots, a combination with other actuating methods (such as magnetic fields) could be beneficial [143]. In addition, simple open-loop controls have been prominently reported. Future research can focus on feedback controllability that allows precise manipulation of such robots to the targeted area. In addition, more complicated environments should be tested to demonstrate meaningful biomedical applicability. Real-time tracking and visualization of microrobots in vivo also remain challenging and are open for investigation. Finally, the design of MNRs remains simplistic, the biodegradability and biological circulation of these MNRs still remain the limitation in “real” application. Future research can focus on composite metallic materials to enhance the biocompatibility and biological circulation, and developing more complicated robots that can carry therapeutics/imaging agents that allow the robots to perform “real” theranostics functions.

In summary, acoustically actuated MNRs for biomedical applications represent an emergent, multidisciplinary research frontier, encompassing diverse scientific disciplines such as physics, materials science, biology, chemistry, and electronics. Effective collaboration plays an important role in multidisciplinary research team success. It involves a combination of shared goals, effective team dynamics, a holistic approach, appropriate training, and overcoming challenges. This collaborative effort ultimately leads to the advancement of knowledge and contributes to the betterment of humanity. In the preceding decade, noteworthy advancements have transpired in biosensors, microfluidic platforms, lab-on-chip technologies, 3D bioprinting, and microelectromechanical systems. These developments furnish a spectrum of potent technologies that contribute significantly to the progress of MNRs research field. The non-invasive and biocompatible characteristics inherent in acoustic waves, coupled with their versatility, exceptional spatiotemporal resolution, and considerable potential for customization position them as a robust platform for biomedical application. The integration of miniaturized systems with microfluidics such as lab-on-chip, system-on-chip system or with living cell system such as organ-on-chip, human-on-chip technology, has enabled innovation and scientific discoveries.

## 5. Conclusions

The main purpose of this review was to provide a panoramic view of acoustically actuated MNRs for biomedical applications; from fundamental to state-of-the-art acoustic actuators for the manipulation of MNRs. First, we introduced the actuating method using acoustic waves to manipulate objects, including the principles of operation and the different types of acoustic actuators currently in use. Then, the applications involving manipulating different types of MNRs were reviewed, including bubble-based robots, bubble-free robots, biohybrid microrobots, and nanorobots. Finally, we discussed the challenges and future perspectives for development within the field. With the increasing interest in acoustically actuated MNRs in biomedical applications in recent decades, we strongly believe that this review will provide researchers with a comprehensive overview of controlling MNRs using acoustic actuators in biomedical applications.

## Figures and Tables

**Table 1:** Acoustically actuated MNRs strategy.

Acoustic Actuation Strategy	Propelled Mechanism	Acoustic Control Type	Application
Acoustic tweezer	Standing-wave tweezers	Surface acoustic wave [4,39,54,55,56,57] Bulk acoustic wave [58,59]	Nanorobots manipulation cell delivery cell separation or patterning Levitation of cells and submillimeter organisms
	Traveling-wave tweezers	Acoustic phase modulation [60,61,62] Acoustic lens [63,64]	3D translation and rotation of MNRs high-resolution ultrasonic imaging bioprinting and tissue engineering Targeted drug delivery
Streaming-driven acoustic	Bubble oscillation	Tubular shape [50,65,66,67] Cup shaped [68,69]	3D translation and rotation of MNRs Targeted drug delivery
	Geometric design	Axis symmetric shape [70,71] Flagellar swimmer [72,73]	Cell separation or patterning Targeted drug delivery Sensing

**Table 2:** Manipulation of different types of acoustic actuators for manipulation of MNRs and specific applications.

No.	Acoustic Actuator Type, Operating Frequency	Robot Type	Applications	Ref.
1	30 ultrasound transducer array, active traveling waves, 1 MHz	Nanorobot	3D manipulation	[17]
2	Piezoelectric disk transducer, 237 kHz	Bubble-based microrobot	3D manipulation	[39]
3	Ultrasonic 16-transducer array, active traveling wave, 1 MHz	Bubble-free microrobot	3D manipulation, targeted drug delivery	[62]
4	Piezoelectric transducer, 4.6 kHz	Bubble-free microrobot	Remote actuation	[71]
5	64-element array acoustic tweezer, 3 MHz	Bubble-based microrobot	In vivo tumor targeting	[116]
6	Piezo transducer, 490 kHz	Bubble-based microrobot	In vivo manipulation	[117]
7	Piezoelectric transducer, 70–270 kHz	Bubble-based microrobot	Multiple DoF locomotion, cancer cell lysing	[118]
8	PA1951 transducer, 50–120 kHz	Bubble-based microrobot	Debris clearance, cell collection	[119]
9	Ceramic piezoelectric transducer, 320 kHz	Bubble-based microrobot	Epithelial pinning and drug delivery	[122]
10	Piezo transducer, 12−19 kHz	Bubble-free microrobot	2D, 3D manipulation	[123]
11	Piezo transducer, 5–270 kHz	Bubble-free microrobot	Biomanipulation, targeted therapy	[124]
12	Focused US transducer, 500 kHz and 2 MHz	Bubble-free microrobot	Active cell tagging, navigation, and US imaging	[125]
13	64-element array acoustic tweezer, 3 MHz,	Biohybrid microrobot	In vivo manipulation, anticancer therapy	[127]
14	Piezoelectric transducer, 2.93 MHz	Biohybrid microrobot	Therapeutic transport	[133]
15	30 ultrasound transducer array, active traveling waves, 1 MHz	Biohybrid microrobot	3D manipulation, targeted drug delivery	[134]
16	Piezoelectric transducer, 618 kHz, 2.66 MHz	Nanorobot	Autonomous reconfiguring operation	[135]
17	Piezo-actuator, 4.6 kHz	Bubble-based microrobot	3D maneuverability	[136]
18	Immersion ultrasound transducer, 234 kHz, 301 kHz	Bubble-based microrobot	Targeted drug delivery, remote microsurgery	[137]
19	Piezoelectric transducer, 28.0 kHz	Bubble-free microrobot	Acousto-magnetic manipulation	[138]
20	Piezo transducer, 20–100 kHz	Bubble-free microrobot	Analogous microparticle trap	[139]
21	Piezo transducer, 22.3−23 kHz	Bubble-based microrobot	Train-like assembly and cargo transport	[140]
22	Piezo transducer, 4.25 MHz	Bubble-based microrobot	In vivo manipulation	[141]
23	Piezo ceramic transducer, 1 to 3 MHz	Bubble-based microrobot	Single-particle manipulation	[142]

## Data Availability

Not applicable.
